# Perspective of Micro Process Engineering for Thermal Food Treatment

**DOI:** 10.3389/fnut.2018.00024

**Published:** 2018-04-09

**Authors:** Alexander Mathys

**Affiliations:** Sustainable Food Processing Laboratory, Institute of Food, Nutrition and Health (IFNH), ETH Zurich, Zurich, Switzerland

**Keywords:** micro process engineering, thermal processing, food processing, preservation, thermal inactivation, rapid heating, thermal pasteurization, thermal sterilization

## Abstract

Micro process engineering as a process synthesis and intensification tool enables an ultra-short thermal treatment of foods within milliseconds (ms) using very high surface-area-to-volume ratios. The innovative application of ultra-short pasteurization and sterilization at high temperatures, but with holding times within the range of ms would allow the preservation of liquid foods with higher qualities, thereby avoiding many unwanted reactions with different temperature–time characteristics. Process challenges, such as fouling, clogging, and potential temperature gradients during such conditions need to be assessed on a case by case basis and optimized accordingly. Owing to the modularity, flexibility, and continuous operation of micro process engineering, thermal processes from the lab to the pilot and industrial scales can be more effectively upscaled. A case study on thermal inactivation demonstrated the feasibility of transferring lab results to the pilot scale. It was shown that micro process engineering applications in thermal food treatment may be relevant to both research and industrial operations. Scaling of micro structured devices is made possible through the use of numbering-up approaches; however, reduced investment costs and a hygienic design must be assured.

## Introduction

Micro process engineering as tool of process intensification is an emerging and growing field in the chemical engineering domain (1–3); however, knowledge transfer and applications into food engineering have been limited thus far. Process intensification was defined by Stankiewicz and Moulijn (4) as any engineering development that leads to a substantially cleaner, smaller, safer, and more energy efficient technology. According to the process synthesis strategy (5), micro process engineering is a useful approach to adjust a process to a reaction rather than adapting physicochemical phenomena to the process. Micro process engineering uses microfabrication technologies for excellent heat and mass transfers within continuous flow systems. Micro-structured devices contain flow areas with at least one dimension in the lateral direction for flows smaller than several mm (DIN EN ISO 10991). These dimensions lead to extreme surface-area-to-volume ratios of between 10,000 and 50,000 m^2^/m^3^, which are approximately 100 times higher than for conventional equipment (6–8). This technology platform enables more efficient unit operations with more precise process control, higher yields, and a smaller infrastructure associated with more compact processes, and, therefore, modular, scalable, and safer systems for emerging engineering challenges (1–3). The equipment portfolio ranges mainly from several mechanical and thermal process devices, such as micro mixers, heat exchangers, retention volume reactors, combined mixers, and heat exchangers, separators, and several types of peripheral pumps, sensors, and actuators (1–3, 8). These devices can be assembled quite often in a modular way, and such systems enable a fast and flexible process development.

However, the complexity of food matrices, hygienic designs with the related cleaning/sterilization in place (CIP/SIP) requirements, high investment costs, and other challenges have prevented the implementation in the food industry thus far. Roos, Fryer et al. (9) published a review article on multiple scales in the area of food structure engineering, where micro process engineering principles were also considered. The main focus has been on mechanical units for food structure generation using micro mixers and/or micro emulsification devices. A simultaneous emulsification and mixing system combines both process principles within one disruption system. This concept can also be suggested for food applications, such as the emulsification and mixing of high fat dairy cream (up to 42% fat) (10). However, beyond the benefits of such focused mechanical-based mass transport, only very limited documentation exists around thermal food process development focusing on a rapid heat transfer enabled through micro process engineering.

Relevant benefits for the food industry may include (1) innovative temperature–time conditions beyond ultra-high temperature (UHT) process windows, and (2) precise process control, which enables rapid heating and cooling with isothermal dwell times for the reproducible up-scaling of different product volumes from the lab to the pilot and industrial scales. These potential applications are relevant to food research, as well as industrial operations, based on numbering-up approaches to scale mass flows through the production system (2, 3).

This perspective on micro process engineering for thermal food process development gathers the basic principles, promising applications, and potential use in the food industry that is also applicable in the biotechnological and pharmaceutical sectors.

## Micro Process Engineering for Thermal Food Treatment

### Thermal Processing in Micro Structured Devices

Micro process engineering operates at extreme surface-area-to-volume ratios, and owing to the respective laminar flow conditions in micro structured heat exchangers (6, 11), the main relevant mechanism of a heat transfer is thermal conduction (8). Typical Reynold’s numbers are within the range of 10–500 (6). Recent developments integrated microstructures into heat exchangers and a combined mixing during heating enables significant forced convection (12, 13).

The overall heat transfer is defined as the flow of heat between two moving media. It involves heat convection and conduction processes. In a continuous process the rate of heat flow $\dot{Q}$ (W) through the system can be expressed as

(1) $$\dot{Q}=A\cdot U\Delta \vartheta_{m}=\dot{V}\rho c_{p}\left(T_{o}-T_{i}\right),$$

with contact area for each fluid side *A* (m^2^), overall heat transfer coefficient *U* (W m^−2^ K^−1^), mean logarithmic temperature difference Δ*ϑ_m_* (K) between the two flows, flow rate $\dot{V}$ (m^3^ s^−1^), density ρ (kg m^−3^), specific heat capacity *c_p_* (J kg^−1^ K^−1^), and inner and outer temperature *T_i,o_* (K).

If the reactor residence time τ (s), volume *V* (m^3^), and respective flow rate $\dot{V}$ are combined with Eq. 1, it becomes visible that the volume-based heat transfer capability $\frac{V}{A\cdot U}$ determines the heating or cooling time, as shown in Eq. 2,

(2) $$\tau =\frac{V}{\dot{V}}=\frac{V}{A\cdot U}\frac{\rho c_{p}\left(T_{o}-T_{i}\right)}{\Delta \vartheta_{m}}.$$

Extreme surface-area-to-volume ratios can lead to very low process times in this perspective.

Thermal conduction can be expressed using Fourier’s law in Eq. 3, where the local heat flux density $\vec{q}$ (W m^−2^) is equal to the product of thermal conductivity λ (W m^−1^ K^−1^), and the negative local temperature gradient, −∇T (K m^−1^).

(3) $$\vec{q}=-\lambda \nabla T$$

During steady-state conduction in a pipe or cylinder with inner and outer radius *r_i,o_* (m), and length *l* (m), the rate of heat flow $\dot{Q}$ through the cross-sectional area in Eq. 4 is described as

(4) $$\dot{Q}=2\pi l\lambda \frac{T_{i}-T_{o}}{ln\frac{r_{o}}{r_{i}}}.$$

The thermal diffusivity *a* (m^2^ s^−1^) in Eq. 5 characterizes the rate of heat transfer of a material from the hot side to the cold side,

(5) $$a=\frac{\lambda }{\rho }c_{p}.$$

In this respect, the characteristic time *t*_characteristic_ for thermal diffusion, which is the typical time it takes for heat to be transported over a particular distance, shows a quadratic dependency on the characteristic length in the lateral direction, as shown in Eq. 6.

(6) $$t_{\text{characteristic}}=\frac{r^{2}}{a}.$$

Based on this theoretical background of the thermal conduction, micro structured heat exchangers can be sufficiently compared with conventional systems in terms of the heat transfer performance. Freund and Sundmacher (8) discussed this comparison using data for a capillary with a circular cross-section (6). They concluded that the characteristic time *t*_characteristic_ for thermal conduction using typical values for thermal diffusivity *a* of liquid water (1.5 × 10^−7^ m^2^ s^−1^) does not reach more than 1 s until radius dimensions of approximately 400 µm are considered, as shown in Eq. 6, under standard pressure and temperature conditions. Calculations with the same boundary conditions showed that a radius of 10 µm results in a *t*_characteristic_ of 0.67 milliseconds (ms) and 100 µm results in a *t*_characteristic_ of 66.6 ms (6). Furthermore, Freund and Sundmacher (8) concluded that the overall heat transfer can be increased by a factor of 1,000, if compared to heat exchangers of a few cm in diameter.

These data demonstrate the strong potential of micro structured heat exchangers to overcome limiting steps in the heat transfer performance and process intensification.

During thermal food processing applied for preservation purposes, the heat transfer performance and residence time distribution (RTD) during the heating and holding periods are important. Minimal residence times are safety relevant, and maximal residence times can lead to losses in quality. This behavior is also relevant during CIP, SIP, crystallization, and drying (14). Therefore, an exact understanding of RTDs in micro structured devices is necessary and needs to be assured (11). Because the integral of the temperature–time profile leads to the applied thermal intensity, process optimization, and product development are also based on narrow RTDs in combination with an optimized heat transfer performance.

### Thermal Degradation of Bacteria and Food Compounds

Thermal degradation during food processing is typically quantified based on a decadic logarithm of the concentration changes under isothermal conditions, as indicated in Eq. 7:

(7) $${\text{log}}_{10}\left(\frac{C{\left(t\right)}_{T}}{C_{0}}\right)=-\frac{k_{T}}{2.303}.\;t,$$

where the initial concentration is *C*_0_, the concentration during different holding times is *C*(*t*)*_T_*, the velocity rate constant is *k_T_* (s^−1^), and the isothermal holding time is *t* (s). This relationship enables the calculation of the *D_T_*-value (decimal reduction time), which represents the time necessary at a specific temperature to reduce the concentration to 1/10 of the original value, as shown in Eq. 8.

(8) $$D_{T}=\frac{2.303}{k_{T}}.$$

The temperature dependency of the velocity rate constant *k_T_*, and thus of the *D_T_*-value, during the processing is given by Arrhenius (15), as shown in Eq. 9:

(9) $$\text{ln}\left(\frac{k_{T}}{k_{0}}\right)=-\frac{E_{a}}{R}.\frac{1}{T},$$

where the energy of activation is *E_a_* (J mol^−1^), the universal gas constant is *R* (8.314 J mol^−1^ K^−1^), and the velocity constant is *k*_0_ for 1/*T* = 0.

The Arrhenius equation enables the determination of the energy of activation *E_a_*, which in combination with the *D_T_*-value sufficiently characterizes the thermal degradation of bacteria or food compounds.

During real processing, non-isothermal conditions occur with a change in *D_T_*, and the dimensionless death value represents the inactivation by the fully applied thermal process intensity, shown in Eq. 10.

(10) $$\text{Death value}=\int_{0}^{t}\frac{dt}{D_{T}\left(t\right)}$$

The inactivation of 10^9^ thermophilic spores per gram (−9 log_10_ g^−1^; death value of 9) is considered an accepted sterilization condition (14). Another approach must feature the 12 × *D*_121.1°C_ concept to guarantee commercial sterility of the product (16), which represents the required process intensity (12 × *D*_121.1°C_ 0.21 min = 2.52 min at 121.1°C) for inactivation of *Clostridium botulinum* spores in canned foods. Both approaches consider only first-order linear log_10_ reductions (14), and cannot cover a broad range of observed inactivation curves, such as shoulder and tailing formations (14, 17–21). However, both concepts are successfully applied in food preservation, mainly by including safety hurdles, such as the consideration of very high initial microbial counts before treatment, which might not be realistic.

### New Temperature–Time Conditions Beyond Conventional Thermal Process Windows

**Figure 1:**
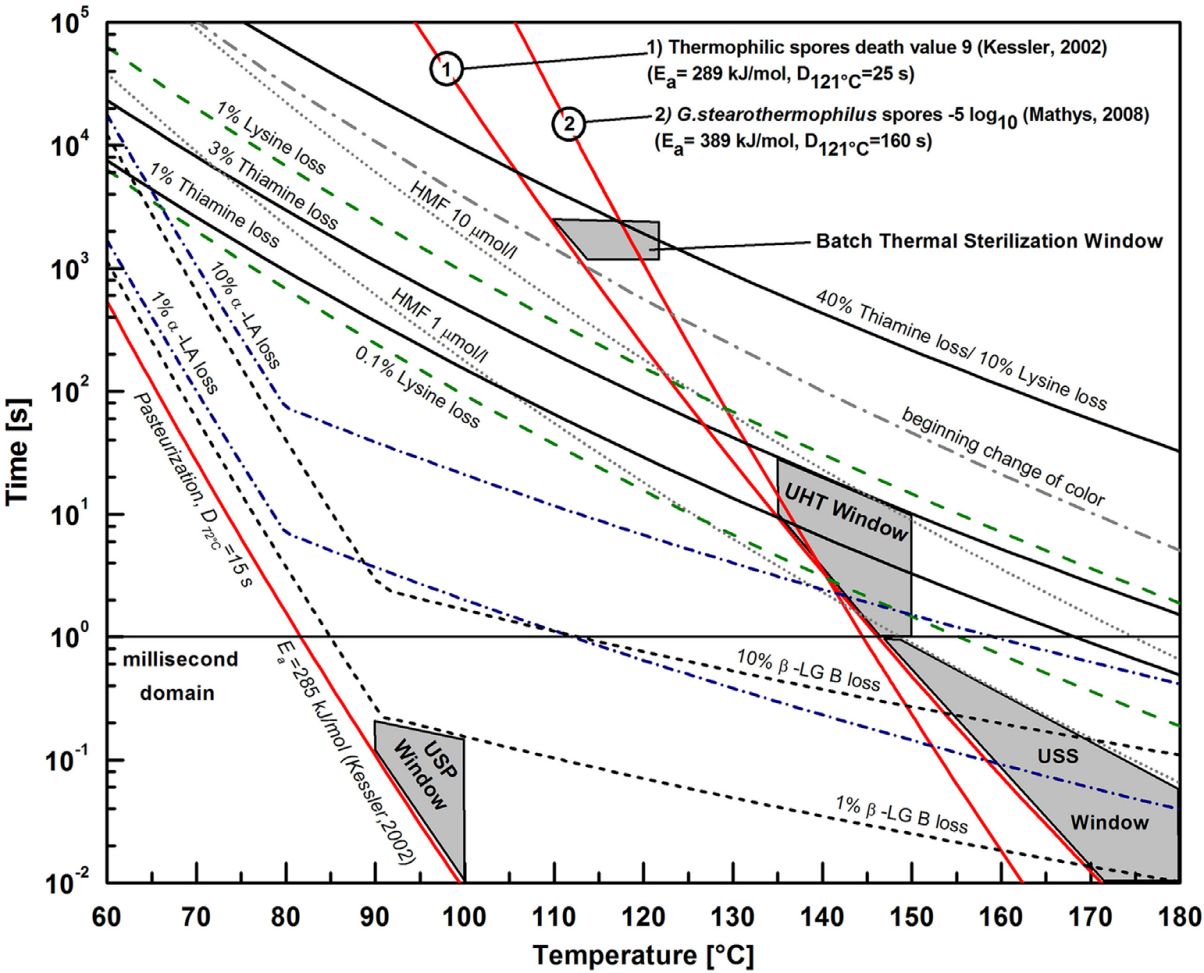
Characterization of process windows for thermal preservation (pasteurization and sterilization) with respective wanted microbial and unwanted biochemical effects with milk as the model system, as modified from Kessler (14). Process windows above 1 s belong to conventional treatments and process windows below 1 s (ultra-short pasteurization and ultra-short sterilization) can be used as new treatments based on micro structured reaction systems.

The thermal processing of food includes a broad spectrum of operations, mainly grouped as processes focused on the heat or mass transfer. Processes focused on heat transfer can be distinguished with or without a phase transition, where the last group, includes heating, holding, and cooling. This group is the focus of Figure 1, where thermal preservation (pasteurization and sterilization) with the respective wanted microbial, and unwanted biochemical effects with milk as the model system are presented. Unwanted biochemical effects in milk also include hydroxymethylfurfural (HMF) generation, β-Lactoglobulin B (β-LG B) loss, and α-Lactalbumin (α-LA) losses. All other reactions are described in Figure 1.

Figure 1 depicts the temperature–time relationships of different kinetic data in milk described in the literature (14). Kinetics with temperatures over 160°C and times less than 1 s are not validated. The different mathematical and theoretical backgrounds are given in Eqs 7–10. The temperature–time relationships are calculated based on the Arrhenius equation in Eq. 9. All inactivation data on microorganisms with their respective *D_T_* and *E_a_* values represent wanted reactions (pasteurization or sterilization), whereas additional data (straight line Nr. 2) from Mathys (22) were included to demonstrate variations in the process objective (commercial sterility). Mathys (22) used a certified liquid sterilization indicator, namely, *Geobacillus stearothermophilus* ATCC 7953 spores (NAMSA, OH, USA; certified by steam *D*_121°C, Steam_ = 144 s, *E_a_* = 409 kJ mol^−1^), which were inactivated in thin glass capillaries, as described elsewhere (19). The inactivation line (straight line Nr. 2) represents a reduction of 5 log_10_ in ACES buffer (pH 7), whereas the inactivation line of the thermophilic spores (straight line Nr. 1) based on Kessler (14) characterizes a death value of 9, as shown in Eq. 10. A death value of 9 represents the inactivation of 10^9^ thermophilic spores per g (−9 log_10_) based on the fully applied thermal process intensity, including the non-isothermal conditions. This process objective line (straight line Nr. 1) defines the windows for batch sterilization and UHT (14), where data for a 5 log_10_ reduction of *Geobacillus stearothermophilus* spores (straight line Nr. 2) run through the batch sterilization and partially through the UHT process window. This behavior demonstrates the limitations of conventional thermal sterilization process windows, which also depend on the respective initial concentration, matrix conditions, and overall thermal intensity.

Emerging process windows using a micro structured reaction system with a very high heat transfer performance can be suggested based on optimizations between wanted and unwanted reactions within the ultra-short process domain in Figure 1 (t < 1 s). These emerging thermal preservation process windows below 1 s can be defined as ultra-short pasteurization (USP) and ultra-short sterilization (USS), where USP is within the range of 10–200 ms at 90–100°C, and USS is between 10 ms and 1 s at 147–180°C. Because the suggested process windows are defined as below certain respective quality relevant kinetics, these reactions can be avoided while reaching the respective process objective (pasteurization or sterilization), thereby leading to a higher quality product with similar safety conditions. However, quality is a broad term consisting of organoleptic, nutritional, and health related properties of food, where Figure 1 could only present examples in milk. In this case, nutritional and health-related reactions (1–10% β-LG B loss, 1% thiamine loss, 0.1% lysine loss, 1–10% α-LA loss, 1 µmol l^−1^ HMF generation) could be avoided by applying innovative USP or USS, respectively. Based on these relationships, it could be assumed that organoleptic properties in milk are also enhanced. However, it is not clear whether these organoleptic benefits could be recognized by consumers. One fundamental condition for possible quality benefits is the significant difference of the *E_a_* between wanted (*E_a_*↑) and unwanted reactions (*E_a_*↓).

The idea of ultra-short thermal preservation or inactivation has already been considered in other peer-reviewed publications (22–25) and patent applications (26–28). However, no systematic overview of food applications under conditions applied within the millisecond range has yet been developed.

Other research groups have discussed the use of extreme short thermal effects combined with ultra-high-pressure technology (29–33). Mathys, Reineke et al. (32) designed a heating–cooling block around an ultra-high-pressure capillary of up to 1,200 MPa to realize extreme heat transfers during the pressure build-up phases. The application of continuous ultra-high-pressure homogenization of up to 350 MPa also includes ultra-high-temperatures and ultra-short treatments of around 240 ms (30) in combination with dynamic pressure and cavitation. However, it was summarized that the ultra-high-temperature–time combination might have a dominant role in the lethal dose of the applied sterilization indicators (29, 30). A review of ultra-short-pressure supported thermal inactivation is given by Sevenich and Mathys (33).

However, for indirect heating principles, certain challenges need to be considered. The benefits around a high surface-area-to-volume in micro structured devices can also lead to fouling, clogging, and temperature gradients owing to pressure losses of flows passing through the microstructures. Therefore, such devices and micro process lines need to be evaluated individually to fulfill the needs of continuous food production, such as operational robustness, hygienic design, SIP, and CIP.

### Scale-Up of Thermal Inactivation Processes

**Figure 2:**
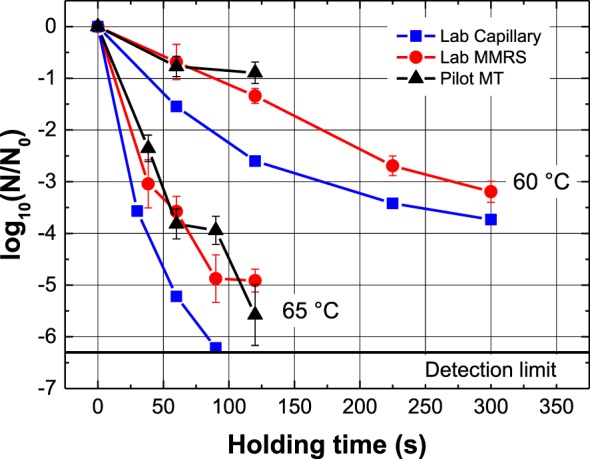
Scale-up case study, inactivation of thermal indicator (*Lactobacillus* mixture in phosphate buffered saline, pH 7.2) during different processes and scales; batch glass capillary of 60 µl (■), continuous modular micro reaction system with a max of 1 kg h^−1^ (●), and a continuous pilot scale (MT) with a max of 75 kg h^−1^ (▲) (34).

The scalability of food research results and process development is a significant challenge for implementation initiatives. The scale-up of thermal inactivation is relevant for food preservation with the investigation of wanted (safety) and unwanted (quality loss) reactions. However, product and process development is quite often challenged based on the transfer of batch lab research results into different mass and heat transfer dimensions, such as continuous pilot and industrial scale processing. Micro process engineering can support this scale-up, as indicated based on data from Mathys, Georget et al. (34), shown in Figure 2.

A continuous modular micro reaction system (MMRS) (Ehrfeld Mikrotechnik GmbH, Wendelsheim, Germany) (11) with a max of 1 kg h^−1^ was compared with a continuous pilot-scale system (MT) (MicroThermics, Inc., Raleigh, NC, USA) with a max of 75 kg h^−1^ and typically used batch lab-scale glass capillaries with 60 µl, described elsewhere (19). A spray-dried thermal indicator (*Lactobacillus* mixture, Nestlé Research Center, Lausanne, Switzerland) was reconstituted in Dulbecco’s phosphate buffered saline (PBS) (Sigma-Aldrich Company, LTO Irvine, UK; pH 7.2) with an initial concentration *C*_0_ of 10^8^ CFU per g. Inactivation was analyzed by plate count on MRS agar in triplicates (34).

Two different treatment temperatures (60, 65°C) were selected to compare the inactivation kinetics (Figure 2). The kinetics were comparable between 0 and 150 s; however, the pilot-scale MT pump was limited, and longer holding times at 60°C could not be compared between the MMRS and MT.

The kinetics for the two continuous systems (MMRS and MT) are close to the batch values, obtained using glass capillaries. It should be noted, however, that the kinetics observed with the MT and the MMRS may correspond to a first-order inactivation kinetic, whereas the capillary experiments might suggest a higher order (14). For an absolute comparison of the MMRS and MT systems, first-order kinetic modeling of the data can be used to determine the relevant kinetic parameters (capillary: *D*_60°C_ = 69.2 s, *D*_65°C_ = 12.9 s, *E_a_* = 315 kJ mol^−1^; MMRS: *D*_60°C_ = 88.8 s, *D*_65°C_ = 17.2 s, *E_a_* = 308 kJ mol^−1^, MT: *D*_60°C_ = 134 s, *D*_65°C_ = 19.4 s, *E_a_* = 363 kJ mol^−1^) (34).

These values are comparable, considering the limited data for pilot scale MT, and can also confirm the values found in the literature for other bacteria (14), particularly of the *Lactobacillus* genus for similar temperatures (35, 36).

Overall, the continuous MMRS system seems to be sufficient for an evaluation of thermal impacts on different microorganisms, enzymes, and nutrients for research at continuous lab scale. Furthermore, the potential of continuous lab-scale production, modularity, and a fast switching of the samples, as well as simple aseptic filling using sterile filters with collection bottles has benefits. Georget, Sauvageat et al. (11) investigated the RTDs of the applied MMRS retention reactor and coaxial heat exchanger. The authors found that the mean residence time of the retention reactor was close to the hydraulic residence time, and no major defects in the flow could be considered. However, the RTDs of the MT system have yet to be characterized, which is necessary to finally compare all of the results obtained.

## Conclusion

Micro process engineering as a tool of process synthesis and intensification is a useful approach to adjusting a process to the reaction rather than adapting different phenomena to the process. Limiting steps in the heat transfer performance can be avoided through extreme surface-area-to-volume ratios, which enables ultra-short thermal treatment of foods within the millisecond range.

Innovative pasteurization and sterilization with higher temperatures, but holding times within the millisecond range allow the preservation of liquid foods with higher qualities because several unwanted reactions with different temperature–time characteristics can be avoided. However, the benefits of a high surface-area-to-volume ratio have certain drawbacks, particularly fouling, clogging, and potential temperature gradients that need to be assessed on a case by case basis and accordingly optimized.

Further applications include a more effective upscaling of the thermal processes from the lab to the pilot and industrial scales. It was demonstrated that batch and continuous lab-scale studies of thermal inactivation kinetics can be sufficiently upscaled. Continuous lab-scale studies can significantly improve this procedure by using a greater sample volume than the batch, process conditions more comparable to the pilot and industrial scales, and equipment with easier handling than pilot-scale equipment owing to the respective volume needs.

Micro process engineering applications in thermal food treatment can be relevant in future research and industrial operations. Scaling of micro structured devices is possible through the use of numbering-up approaches; however, reduced investment costs and a hygienic design must be assured.

## Author Contributions

AM would be the only author, summarizing the state of the art, the joined R&D on the topic, and some personal insights on micro process engineering for thermal food treatment.

## Conflict of Interest Statement

The author declares that the present research was conducted with support of the Nestlé Research Center Lausanne, Switzerland; Nestlé PTC Singen, Germany; Ehrfeld Mikrotechnik GmbH, Germany; and ETH Zürich Foundation, Switzerland.
